# Supplementary material for: Identification of photoperiod-induced specific miRNAs in the adrenal glands of Sunite sheep (Ovis aries)
Source: Front Vet Sci. 2022 Jul 22;9:888207. doi: 10.3389/fvets.2022.888207 (PMC9354845; doi:10.3389/fvets.2022.888207)
Supplement: Supplementary file 1 [file Data_Sheet_1.ZIP › Supplementary file/miRDeep2 file.zip!/miRDeep2 file/Novel_Structure/SPLP42A3/Novel_530.pdf]

Provisional ID	:	NC_019466.2_17729
Score total	:	27.2
Score for star read(s)	:	3.9
Score for read counts	:	20
Score for mfe	:	1.7
Score for randfold	:	1.6
Score for cons. seed	:	
Total read count	:	51
Mature read count	:	47
Loop read count	:	0
Star read count	:	4

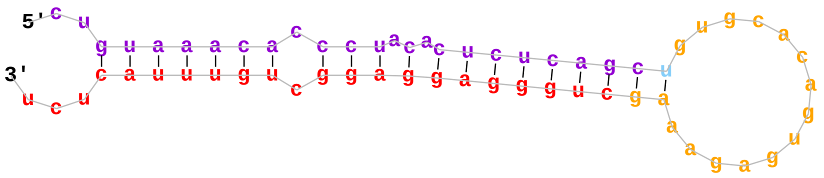

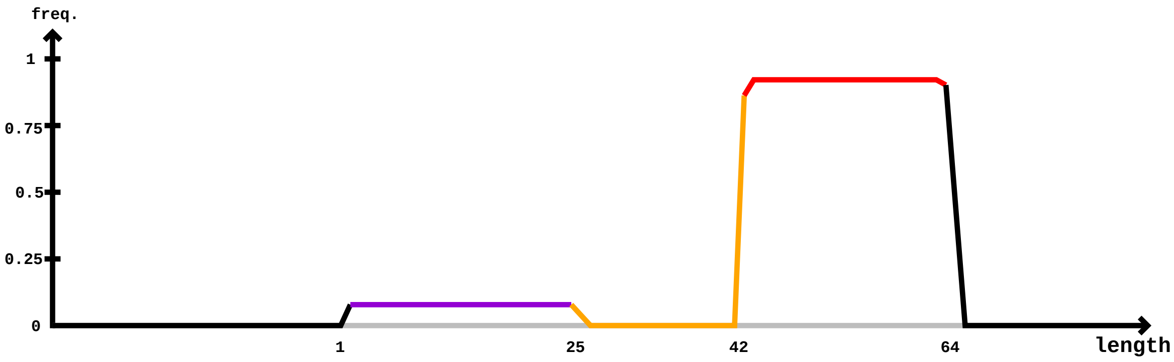


```
                                  Star                                          Mature
5'- ccgccccagagagcacugagggccagauacuguaaacacccuacacucucagcugugcacagugagaaagcugggaggaggcuguuuacucucucugccuuggaagucaacu   -3'   obs
    ccgccccagagagcacugagggccagauacuguaaacacccuacacucucagcugugcacagugagaaagcugggaggaggcuguuuacucucucugccuuggaagucaacu         exp
    ..(((((((......))).))))((((....(((((((.(((.(.(((((((((..............))))))))))))).)))))))....))))...............   reads   mm   sample
    .............................cuguaaacacccuacacucucagc...........................................................   4       0    seq
    ......................................................................cugggaggaggcuguuuacuc.....................   1       0    seq
    ......................................................................cugggaggaggcuguuuacucu....................   41      0    seq
    ......................................................................cugggaggaggcuguuuacucuc...................   2       0    seq
    .......................................................................ugggaggaggcuguuuacucu....................   2       0    seq
    .......................................................................ugggaggaggcuguuuacucuc...................   1       0    seq
```
